# Geographic variation in abundance and diversity of *Acinetobacter baumannii Vieuvirus* bacteriophages

**DOI:** 10.3389/fmicb.2025.1522711

**Published:** 2025-01-28

**Authors:** Dafne Arellano-Maciel, Juan Manuel Hurtado-Ramírez, Laura Carolina Camelo-Valera, Santiago Castillo-Ramírez, Alejandro Reyes, Gamaliel López-Leal

**Affiliations:** ^1^Laboratorio de Biología Computacional y Virómica Integrativa, Centro de Investigación en Dinámica Celular, Universidad Autónoma del Estado de Morelos, Cuernavaca, Mexico; ^2^Instituto de Biotecnología, Universidad Nacional Autónoma de México, Cuernavaca, Mexico; ^3^McGill Centre for Microbiome Research, Department of Microbiology and Immunology, McGill University, Montreal, QC, Canada; ^4^Programa de Genómica Evolutiva, Centro de Ciencias Genómicas, Universidad Nacional Autónoma de México, Cuernavaca, Mexico; ^5^Grupo de Biología Computacional y Ecología Microbiana, Departamento de Ciencias Biológicas, Universidad de los Andes, Bogotá, Colombia

**Keywords:** prophages, bacteriophages, *Acinetobacter baumannii*, *Vieuvirus*, phages

## Abstract

**Introduction:**

Prophages play a crucial role in the genomic diversity of *Acinetobacter baumannii*, contributing to its pathogenicity and adaptation.

**Methods:**

In this study, we induced and sequenced seven prophages from five isolates of *A. baumannii*. These were analyzed with 967 prophages identified from various isolates worldwide, plus 21 genomes of other phages infecting *A. baumannii* previously reported in NCBI. To have an overview of the populations of the prophages infecting *A. baumannii*.

**Results:**

Our analysis revealed 13 major prophage clusters within the analyzed *A. baumannii* isolates. Notably, prophages belonging to the *Vieuvirus* genus were the most prevalent. Specifically, *Vieuvirus*-related phages were frequently identified in isolates from Thailand, Mexico, China, and South Korea, which show the geographic prevalence of *A. baumannii* prophages.

**Discussion:**

This study highlights the importance of considering geographic factors to fully understand prophage diversity and their significant role in the evolutionary dynamics of *A. baumannii*.

## Introduction

Viruses are the most abundant biological entities on Earth (Breitbart and Rohwer, 2005). Bacteriophages, or phages, specifically infect prokaryotic microorganisms. These phages replicate either through the lytic cycle, which is typical of virulent phages, or integrate into the host genome as prophages, or replicate as plasmids in the host cytoplasm (Piligrimova et al., 2021). The integrated phage genome (prophage) replicates together with the host chromosome and is transferred vertically from the initial infected cell to its progeny through cell division (Maurice et al., 2013). Integration into the bacterial chromosome can modify the host phenotype and introduce new genes and functions into the bacterial metabolic repertoir (Ramisetty and Sudhakari, 2019). Prophages encode genes responsible for antibiotic resistance and/or virulence factors (Costa et al., 2018; Kondo et al., 2021; López-Leal et al., 2020; Piña-González et al., 2024), confer adaptive benefits to their bacterial hosts (Li et al., 2017; Selva et al., 2009), and facilitate the dissemination of these traits to other microorganisms (Wendling et al., 2021). The growing threat of antimicrobial resistance has emerged as a critical public health issue, with an estimated 700,000 deaths annually attributed to drug-resistant bacterial infections (Myers, 2016; O’Neill, 2014). *Acinetobacter baumannii* is a major cause of nosocomial multidrug-resistant (MDR) infections (Motbainor et al., 2020). It has been identified as a critical target in the World Health Organization’s Priority List for Research and Development of New Antibiotics. Despite the significant knowledge available on the genomics and phylogenomics of *A. baumannii*, studies on phage and prophage populations that infect this species remain scarce. Recently, virulent phages have garnered attention as potential alternative therapies for *A. baumannii* infections and other MDR bacterial infections (Schooley et al., 2017; Wang et al., 2024), particularly for combating hospital-acquired pathogens. However, prophage populations, especially those that could be inducible or potentially active, have received far less attention. Knowing the inducible-prophage populations in target pathogens could help us to understand phage-host dynamics and whether prophages could interfere with phage therapy, as studies have reported recombination events between prophages and virulent phages when infecting their hosts (Piña-González et al., 2024; De Paepe et al., 2014; Dragos et al., 2021), as well as block cell surface receptors to prevent infection by other phages (Chung et al., 2014; Mcallister and Barrett, 1977). Advances in sequencing technologies and bioinformatics tools have increased considerably in recent years, allowing in-depth exploration of the vastness of prophage diversity (Andrade-Martínez et al., 2022). These tools are limited to only identifying the integrity and quality of prophages. A major limitation in identifying prophages is the certainty to determine whether these prophages are potentially inducible. Therefore, identifying active prophages remains a bioinformatic challenge, resulting in recourse to traditional microbiology.

In 2021, we reported a mitomycin-C-inducible phage of the genus *Vieuvirus* (accession number MT361972) isolated from an MDR *A. baumannii* strain Ab11510 (López-Leal et al., 2021), which belongs to the Sequence Type (ST) 758 lineage (Graña-Miraglia et al., 2017). At that time, very few phages of this genus had been reported in public databases, and only phages Bphi-B1251 (Jeon et al., 2012) and YMC11/11/R3177 (Jeon et al., 2015) were reported as reference Vieuviruses by the International Committee on Taxonomy of Viruses (ICTV). Additionally, exploration of prophage populations in different genomes of *A. baumannii* showed that Bphi-B1251 phages are the most prevalent type of phages in *A. baumannii* (Loh et al., 2020), suggesting that phages of the *Vieuvirus* genus may have high infectivity and a broad host range of active phage particles or that they are the most ancestral and segregate and co-evolve with the population (Loh et al., 2020). A recent study of prophage populations in 1,613 *Acinetobacter baumannii* genomes revealed that most prophage species exhibit a limited host range and are geographically restricted. However, some species are cosmopolitan and highly abundant. Despite these findings, knowledge about prophage populations in a broader genomic context, including lytic phages and inducible prophages in *A. baumannii*, remains limited (Tenorio-Carnalla et al., 2024). To expand the catalogue of inducible phages infecting *A. baumannii*, we isolated seven temperate phages from different MDR strains of *A. baumannii* and analyzed their phylogenetic relationships with 928 prophages collected from other global isolates of *A. baumannii*. This study provides a comprehensive characterization of *A. baumannii* prophage diversity.

## Materials and methods

### Bacteriophage isolation and genome assembly

Bacteriophages, phi9102 (PP898111), phi4197 (PQ432283), phi5013-M1 (PQ432284), phi5013-M2 (PQ432285), phi5038-11536 (PQ432286), phi11547 (PQ432287), phi5038-11551 (PQ432288), all mitomycin-C-inducible prophages were obtained from the clinical MDR *A. baumannii* strains collected from the Instituto Nacional de Cancerología (Mexico’s National Institute of Oncology), a tertiary hospital located in Mexico City. Namely, GCA_004299615.1 (phi5013-M1 and phi5013-M2), GCA_003522845.1 (phi9102), GCA_004321575.1 (phi4197), GCA_004794205.1 (phi5038-11536, phi5038-11551), and GCA_001922695.1 (phi11547). In brief, the strains were treated with mitomycin C at a final concentration of 1 μg/ml in LB broth at 37°C for an overnight incubation period. Subsequently, the supernatants were collected via centrifugation (12,000 × *g* for 10 min) and utilized for host-range assays. We used the previously reported strains to propagate the phages (López-Leal et al., 2021). Specifically, 10 μl of each supernatant fraction was applied to overlay plates containing 3 ml of soft agar, followed by the addition of 300 μl of bacterial cells (López-Leal et al., 2021; Kropinski et al., 2009). After incubating overnight at 37°C, the plates were scrutinized for lysis within the spotted region. Confirmation of plaque formation in susceptible strains was achieved using a previously described method (López-Leal et al., 2021; Hyman, 2019; Santamaria et al., 2014). To summarize, 100 μl of a bacteriophage solution (or a dilution from the stock) was added with 200 μl of previously cultured, susceptible *A. baumannii* cells. The mixture was incubated for 15 min at room temperature, after which it was mixed with soft agar and spread onto LB solid medium to create a bacterial lawn. The plates were incubated at 37°C, and the formation of lytic plaques was observed. Individual plaques were picked and subjected to three consecutive rounds of replating to ensure the purity of the bacteriophage stocks. Different plaque morphologies were also considered for bacteriophage isolation. To ensure reproducibility, all experiments were conducted in triplicate. The bacteriophages were then cultivated in 6-ml cultures of *A. baumannii* strains (GCA_001922705, GCA_001922745, and GCA_001922695) in LB medium (with an OD 620 nm of 0.1), supplemented with 100 μl of each bacteriophage (average of 105 PFUs/ml). DNA isolation procedures followed established protocols (López-Leal et al., 2021; Santamaria et al., 2014), and restriction enzyme digestion analysis was performed using *Hind*III, *EcoR*I, *EcoR*V, *BamH*I, and *Nde*I. Those phages that generated a unique restriction patterns were selected for sequencing. Phage genomic DNA sequencing was performed using a Miseq Illumina platform with a 300-bp paired-end configuration with a TruSeq DNA library. Raw reads were trimmed using TRIM_ GALORE^1^ with a quality threshold of ≥30. The genome assembly was performed using the A5 pipeline (Coil et al., 2015) with a set of ~25,000 (average of 7,215,608 reads per phage genome) randomly selected paired-end reads. Then, CheckV (v1.0.3; Nayfach et al., 2021) was used to validate the quality of each assembly. The completeness was confirmed by Bandage (v0.8.1; Wick et al., 2015), using the .gfa file.

### *Acinetobacter baumannii* genomes used

To put our induced prophages into a genomic context with other *A. baumannii* prophages, we used a previously characterized and reported database of 1,465 *A. baumannii* isolates (Hernandez-Gonzalez et al., 2022). The genomes in this database were determined as high-quality genomes according to CheckM (v1.0.2; Parks et al., 2015). BioSample information for all *A. baumannii* genomes was obtained using efetch from E-utilities (v16.2; Sayers, 2009). Metadata was collected for the following sections: source isolate and isolation site.

### Prophage identification

Prophage predictions were carried out using VirSorter2 (v2.2.4; Guo et al., 2021), and CheckV was used to determine the completeness and quality of phages and prophages sequence. Only phage sequences assigned as High-quality or Complete by checkv-quality or medium-quality were considered for downstream analysis. We followed the publicly available protocol for the validation of the first-instance prophage prediction.^2^ Briefly, the final quality of prophages analyzed by CheckV were validated in a second screening using VirSorter2 (Guo et al., 2021). All (pro)phage genomes were annotated using pharokka (v1.6.1; Bouras et al., 2023).

### Clustering at the genus levels and phylogenetic reconstruction

In the first instance, all prophages and phages (all the *Acinetobacter* phages reported in the NCBI) with at least 80% nucleotide similarity in at least 80% of the genome length were binned by cd-hit (v4.8.1; Fu et al., 2012). Following the parameter proposed by the ICTV (International Committee on Taxonomy of Viruses), this first clustering allowed us to select those phages and prophages that belonged to the same genus (Simmonds et al., 2023; Turner et al., 2021). In this sense, groups (phage genera) with more than five members were selected for further analysis. Then, to construct homologous groups (HG) from the selected bacteriophage genomes, we ran roary (v3.13.0; Page et al., 2015), setting the BLAST search parameters to a length coverage of ≥70% and an amino acid sequence identity of ≥40%. Next, a second clustering was made based on the optimal number of clusters determined by the hierarchical clustering of viruses based on intergenomic distances calculated from their shared protein content (pan-matrix from roary). The optimal number of clusters was determined by the average-silhouette method in R. Subsequently, hierarchical trees were constructed for each cluster with 100 bootstrap replicates. The ape (v5.6.1) and ggtree (v 3.0.4) libraries in R were used to obtain the branch length distribution. Finally, phage species and phage genera were validated using VICTOR (Meier-Kolthoff and Goker, 2017).

### Phage diversity and abundance

The relationship between phage abundance and geographical location was evaluated. First, we calculated the uncertainty coefficient between bacteriophage genera and species against geographic locality. Second, the correlation between the abundance of phage species and phage genus in the different countries was computed with Pearson tests. The phage species and phage genus were taken as the result of the VICTOR analysis (Meier-Kolthoff and Goker, 2017).

## Results

### New inducible phages of the *Vieuvirus* genus

In previous studies, we reported that several ST-758 *A. baumannii* isolates have a high abundance and diversity of prophages (López-Leal et al., 2020). Furthermore, some of these phages belonging to the genus *Vieuvirus* were found to be active, since they could cause infections and generate progeny in other strains of the same lineage (López-Leal et al., 2020; López-Leal et al., 2021). In this study, in order to expand our understanding regarding the induced prophage sequences and the prophage population that infects *A. baumannii*, we induced seven prophages from previously reported isolates (see Methods). The phages genomes of phi9102, phi4197, phi5013-M1, phi5013-M2, phi5038-11536, phi5038-11551, and phi11547 were assembled as single contigs of 52,239 base pairs (bp), 39,359, 42,180, 49,159, 48,056, 38,492 and 25,802 bp, respectively. The assembled genomes were assigned as complete and high-quality genomes according to CheckV. No tRNA or ARGs were identified. All induced prophages were similar to the reported Ab11510-phi phage (López-Leal et al., 2021; Supplementary material 1). Interestingly, strains Ab5013 and Ab5038 harbored two genomically similar phages of the genus *Vieuvirus* (Supplementary material 2).

### Thirteen most abundant putative phage genera in species of *Acinetobacter baumannii*

To obtain a better overview of the seven isolated phages in the context of the *A. baumannii* prophage populations, we used a database with 1,501 high-quality *A. baumannii* genomes, according to CheckM (Parks et al., 2015; Supplementary material 3). In an initial analysis, 4,865 prophages were collected. However, only 967 (19.87%) of the prophage predictions were assigned as high-quality by CheckV (Supplementary material 4). To place the seven induced prophages into a genomic and phylogenomic context relative to the prophage population in *A. baumannii*, only high-quality predictions were used, as this approach was considered to provide more reliable results given that prophages are prone to degradation processes (Bobay et al., 2014).

**Figure 1:**
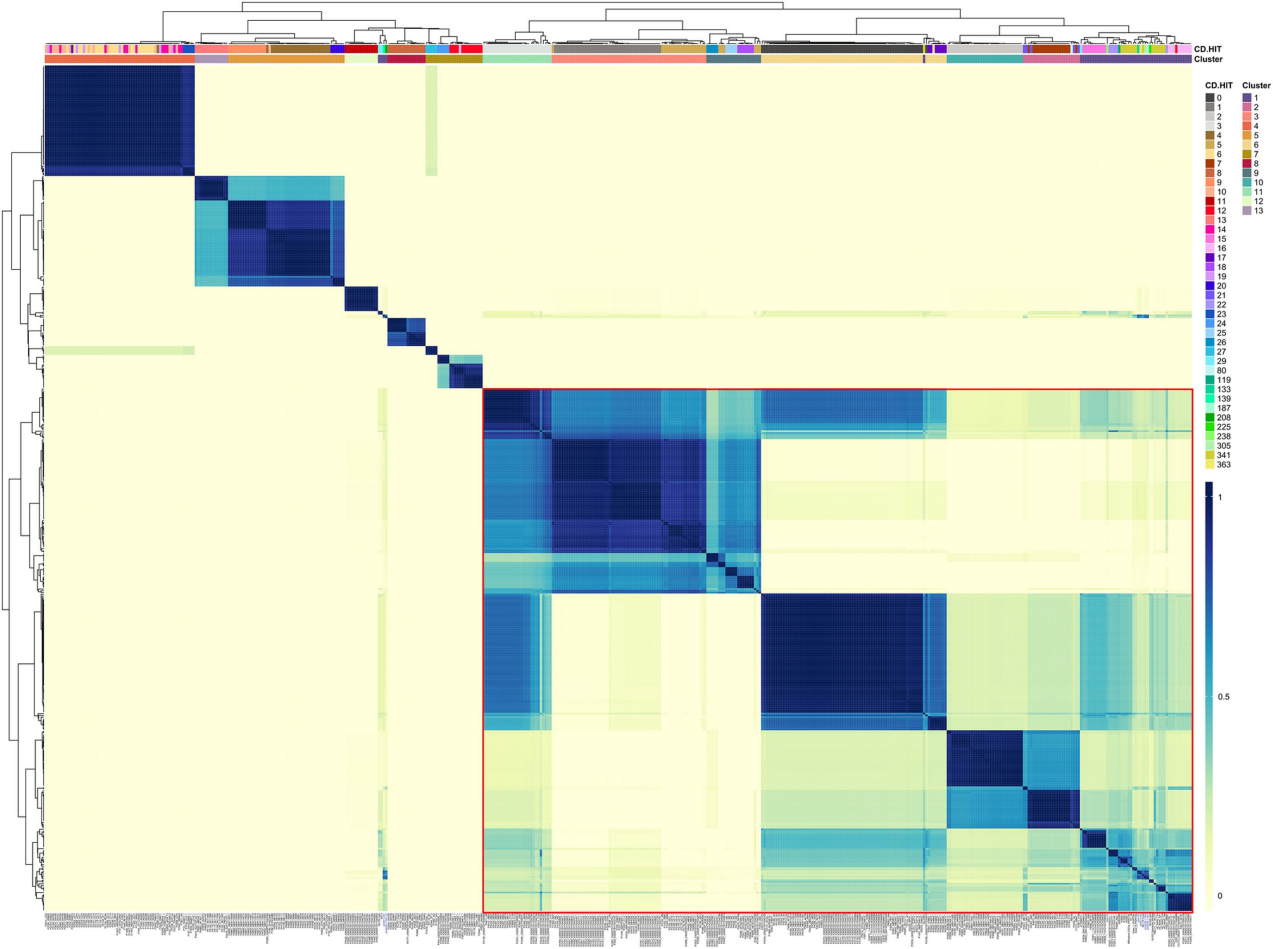
Gene content variation among the phages. A heat map of the gene content correlation matrix was used to analyze the gene content differences among the phages. Clusters according to cd-hit and the average-silhouette (using the correlation matrix) method are indicated in the color bars on the top. The red rectangle detonates those phage groups with the most shared genes.

**Figure 2:**
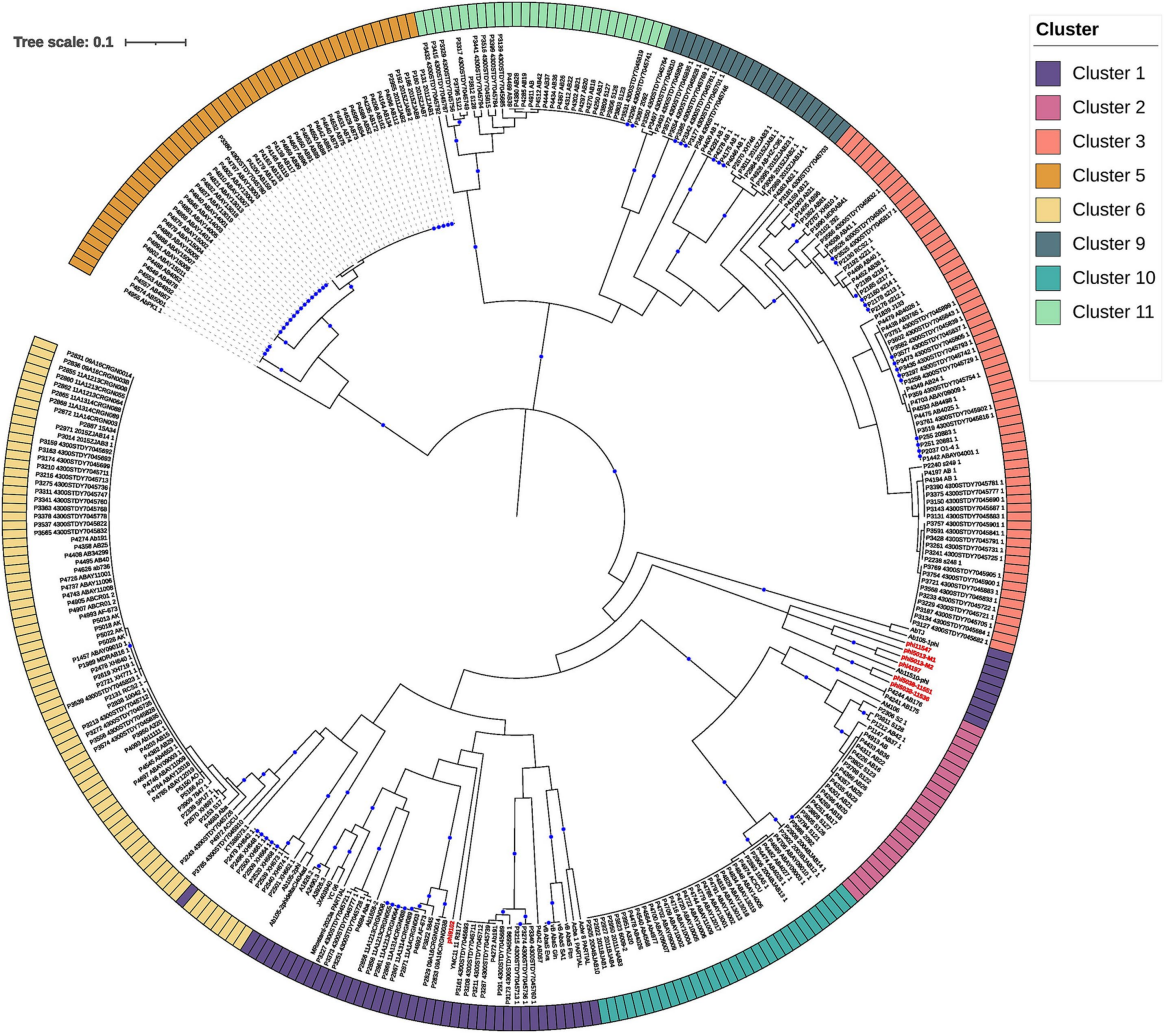
The hierarchical tree was constructed with the PC (protein clusters) from clusters 1, 2, 3, 5, 6, 9, 10, and 11 (rooted using cluster 5; see results) based on intergenomic distances. The external circle provides the clusters, which are designated by the optimal number of clusters, using the average silhouette method (see Figure 1 and Methods section). The bootstrap values higher or equal to 80 are depicted with blue circles at the internal nodes.

These 928 final prophages represented an average of 1.67 prophages per genome. Additionally, we add 21 putative *Vieuvirus* genomes (Supplementary material 5), previously reported (Rastegar et al., 2024), in order to put the induced and isolated prophages (in this study) in context with other previously reported phages of the genus *Vieuvirus* and with prophage populations from *A. baumannii* isolates. Finally, we built an *A. baumannii* phage and prophage genome database with 995 genomes. Of these, we found that 30.02% of phages and prophages were singletons (see Methods), and 69.98% were grouped into 119 clusters using cd-hit. Of these, only phages that clustered with ≥5 others were selected to construct a gene content correlation matrix. This threshold was chosen to focus on the most prevalent phages, which could be grouped into potential genera, providing a clearer understanding of gene content similarity within more broadly distributed phage populations (Figure 1). Furthermore, we observed that the clustering shown in the heat map did not correlate with the groups found in the first level of clustering by cd-hit (see clusters in the top rows of Figure 1). This result suggested that mosaicism of bacteriophage genomes presents challenges to describing phage-relatedness (Gauthier and Hatfull, 2023). In other words, the clustering of gene content (homologous proteins) retrieved by the heat map did not reflect the genome-level clustering obtained by cd-hit. On the other hand, the heat map based on the gene content correlation matrix showed a considerable variation in gene content among the phages, resulting in 13 clusters (50.57% of the phage database). However, we observed some degree of relationship in gene content between phages from clusters 1, 2, 3, 6, and 9–11 (Figure 1, red rectangle). Next, we used the pangenome of the phages to perform a principal component analysis (PCA) based on the pan-matrix. Although only 45.5% of the total variation was captured by the first two components (out of 482 dimensions, Supplementary material 6), the first 10 dimensions accounted for 85.6% of the variance in our data. This suggests that the remaining variation is likely due to the presence of unique genes in each phage, which are not homologous to those in others. Two phage populations were delimited on the first principal (horizontal) axis. Namely, the phages from clusters 4, 5, 7, 8, 12, and 13 were placed within the PC1 component values >0, and the rest were located within the PC1 values <0 (Supplementary material 6). This indicates that the phages of clusters 4, 5, 7, 8, 12, and 13 do not share homologous genes with the rest of the phages or share very few homologous genes. On the contrary, phages from clusters 1, 2, 3, 6, and 9–11 (Figure 1, red rectangle) shared some or several homologous groups (see methods). To further elucidate this, we used a gene content distance matrix to construct a hierarchical clustering tree based on gene content (Figure 2) that only included phages from clusters 1, 2, 3, 6, and 9–11 and rooted the phylogeny using phages from Cluster 5 a closely related genus.

The first thing we noticed is that most of the clades defining the clusters (Figure 2) were well supported (>80 bootstrap values; blue circles). However, clustering boundaries between clusters 1 and 6 had weak support (bootstrap of 45). Interestingly, all members of cluster 6 correspond to prophage predictions, while phages located in cluster 1 correspond to prophage predictions and isolated phages previously reported in the NCBI (assigned as *Vieuvirus*). On this basis, we have tentatively separated these predominantly temperate *A. baumannii* phages into two clusters (Clusters 1 and 6). However, we note that this assignment was poorly supported by the phylogenetic analysis (Figure 2). Interestingly, shorter branches were observed in the Cluster 6 clade (Figure 2). Therefore, we analyzed the distribution of branch lengths for both clusters. Both distributions showed that short branches predominated in both clusters (Supplementary material 7). However, Cluster 6 (Supplementary material 7B) seems to have a higher concentration of extremely short lengths compared to Cluster 1 (Supplementary material 7A), suggesting that the prophages (Cluster 6) have a higher proportion of homolog groups or proteins very similar in sequence so they are even more closely related or have experienced less genetic divergence compared to those in Cluster 1. This indicates that the induced prophages have less HG in common; therefore, they may experience more divergence in their protein repertoire.

Finally, each cluster was validated using the VICTOR tool. Most of the clusters were validated by VICTOR at the genus level, except for phages AbTJ (Xu et al., 2020) and Ab105-1phi, which have been previously reported to share some genetic repertoire with phages of the *Vieuvirus* genus (López-Leal et al., 2021). However, according to the NCBI, these phages are unclassified *Caudoviricetes*. Another exception was for prophages P1576, P1697, P1703, P2118, and P2344 from Cluster 7 (Supplementary material 8), which showed a small degree of similarity with phage AM106 (MH115576), with a coverage of 9–15% and identity of ~90%. In other words, our approach, using the correlation of gene content variation, succeeded in grouping these phages appropriately at the genus level.

### Geographic co-occurrence of bacteriophages reveals countries delimit species

**Figure 3:**
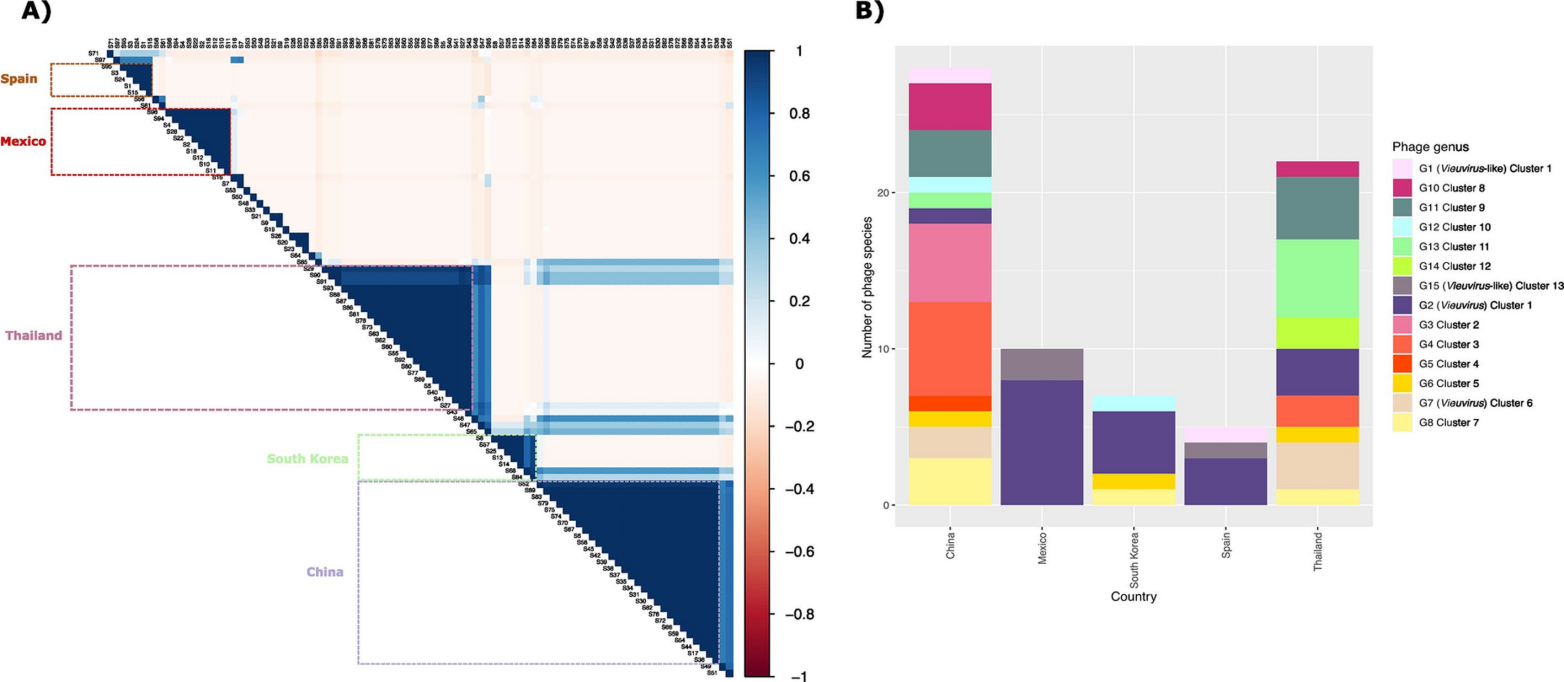
**(A)** Correlation matrix representing Pearson correlation between the relative abundance of species. The color intensity is relative to the correlation coefficients; negative correlations are shown in red, and positive correlations in blue. On the right, the legend shows the corresponding colors and the correlation coefficients. The boxes represent the high correlation species present in each county. **(B)** Bar plot showing the abundance of the 73 phage species by country. The colored panel indicates the genus to which the phage species belongs.

With a total of 482 bacteriophages analyzed (phages and prophages from the 13 clusters), we were able to identify and validate 97 phage species and 15 phage genera (Supplementary material 8). We then determined the sequence type and isolation location of the hosts of the 467 prophages (only prophages from the 13 clusters). This collection of *A. baumannii* isolates comprised 53 different STs (according to Pasteur’s MLST scheme) from 22 countries. Of these, 23.56% of the isolates primarily corresponded to 1806 STs, and 41.69% were from China (Supplementary material 9). Then, in order to identify how widely the bacteriophage genera and species were geographically spread, we first determined the uncertainty coefficient between bacteriophage genera and species. First, we determined the uncertainty coefficient between the genus-species relationships against geographic locality (country) and host ST to determine the relationship strength between these two categorical variables. We found that the mutual information was 1.27 and 0.67, for the species-country and genus-country relationships, respectively (Table 1). These values indicate that the variables share certain information, suggesting a dependence or relationship between them. Specifically, the relationship between species and country is stronger than the relationship between genus and country. Moreover, the residual uncertainty in species was 66.58% after knowing the country, whereas for genus, it was 35.11%. In addition, the residual uncertainty for the country after knowing the genus and species was 24.46 and 31.98%, respectively (Table 1). In other words, residual uncertainty is higher when species or genus is conditioned on country than vice versa, indicating that country has a considerable effect in reducing uncertainty about species or genus. Of these, phage species provide more information about the country and vice versa, compared to genus. With this information, we wanted to see the correlation between the co-occurrence of genera and phage species and the isolation country. A strong positive correlation was observed between the frequency of different species due to their co-occurrence in the same location. Of 73 phage species only located in China (28 species), Thailand (23 species), Mexico (10 species), South Korea (7 species), and Spain (5 species; Figure 3A). Of these, 26.38% of the species corresponded to the *Vieuvirus* genus (G2-Cluster 1; Figure 3B), followed by 11.11% of species for an unclassified genus (G4-Cluster 3), which are similar to the previously reported phage fLi-Aba03 (Rastegar et al., 2024). The rest of the species (with an average of 5.20% of species per genus) belonged to different genera. Interestingly, co-occurrence of phage species is mainly related to the *Vieuvirus* (Figure 3B).

**Table 1:** Relationship between phage abundance and geographic location assessed by the uncertainty coefficient.

X	Y	Mutual Information	U(X|Y)	U(X|Y)_min	U(X|Y)_max	U(Y|X)	U(Y|X)_min	U(Y|X)_max
species	country	1.272894	0.6658364	0.6275773	0.7040955	0.3458607	0.3198087	0.3719127
genus	country	0.6711771	0.3511364	0.3221173	0.3810555	0.2732039	0.2446645	0.3017434
species	host (ST)	1.963887	0.6562884	0.6258662	0.6867107	0.5335639	0.5073782	0.5597496
genus	host (ST)	2.991586	0.4067885	0.3824496	0.4311273	0.4953847	0.4632711	0.5274983

Mutual information and conditional entropy values between bacteriophage genera and species are shown in relation to geographic locality (country) and host sequence type (ST). Mutual information reflects the amount of information shared between the variables. Columns “U(X|Y)” and “U(Y|X)” indicate the uncertainty coefficient, which represents the fraction of uncertainty of X that is reduced by knowing Y, and vice versa, together with their confidence intervals (minimum “min” and maximum “max”).

Although these species belong to different genera, however, it is important to note that phages of genus G2 (Cluster 1; Figure 1) were found more frequently in Thailand, Mexico, Canada, and Iran, while phages of genus G7 (Cluster 6; Figure 1) were found mainly in China, Thailand, Canada, and South Korea (Supplementary material 8). Based on our results (Supplementary material 10), these two groups of phages could be two types of *Vieuvirus* located mainly in North America and South Asia (Cluster 2) and East Asia (Cluster 6). Additionally, the G15 genus, composed of a small number of phages (Cluster 13), was found mainly in isolates from Mexico (Supplementary material 11).

## Discussion

In recent years, the use of bacteriophages as an alternative strategy to antibiotics has gained significant attention. One of the main concerns regarding phage therapy is the evolution of the host genome induced by prophages (Rohde et al., 2018). Recent studies have indicated that pathogenic species accumulate prophage sequences more frequently (Lopez-Leal et al., 2022). Therefore, identifying inducible-prophage populations or potentially active prophages in pathogens such as *A. baumannii* is crucial for studying virus-host relationships.

Here, we analyzed seven inducible prophages from *A. baumannii* combined with 4,671 prophages retrieved from 1,465 *A. baumannii* genomes. Only 19.86% of these predictions were of high quality. We chose to use only high-quality predictions because prophages, once integrated into the chromosome, can become trapped within the host genome due to genomic rearrangements and gradual decay. This domestication of prophages leads to the accumulation of mutations and the loss of genetic material (degradation), which prevents their excision during cell lysis and limits the production of phage particles, as well as lysogenization of non-lysogenic strains (Bobay et al., 2014; Canchaya et al., 2003). These inactive phages are often referred to as cryptic prophages. Although cryptic prophages can also play a role in phage-host dynamics, here, we aimed to explore prophage populations with active potential. On average, we found 1.6 prophages per genome, indicating a substantial prophage presence within this species. It has been reported that species from the genera *Acinetobacter*, *Enterobacter*, and *Pseudomonas* tend to accumulate more prophages (Lopez-Leal et al., 2022).

Additionally, 30.02% of these prophages were found to be singletons, reflecting that more than a quarter of the prophage population in *A. baumannii* are single prophages. Recent studies suggest that this proportion may be even higher. However, Tenorio-Carnalla et al. applied an operational species definition based on ANI values of >95% identity and >90% coverage. Our results revealed that *A. baumannii* prophages exhibit high variability in gene content, likely due to the exchange of genetic material with other phages and their bacterial hosts. This genetic exchange results in genome regions that differ significantly even among closely related phages, potentially causing ANI values to fall below the 95% identity threshold for species definition, despite the phages being functionally similar or closely related in other genomic regions (Tenorio-Carnalla et al., 2024). These observations could be critical to consider if phages that infect these species (especially *A. baumannii*) are to be viewed as an alternative strategy to antibiotic use. In this study, we used the correlation of shared homologous gene content among the phages, which allowed us to group them at the genus level. Notably, these genera were confirmed and validated using the VICTOR tool.

From a phylogenomic perspective, we found that the prophages of *A. baumannii* belong to 13 more prevalent genera, most of which are grouped as unclassified *Caudoviricetes*. However, the *Vieuvirus* genus was among the most abundant in our dataset. These results are consistent with previous studies suggesting a high prevalence of *Vieuvirus*-like prophages in *A. baumannii* (Loh et al., 2020). In this sense, one of the most relevant findings of our study is the identification of two putative *Vieuvirus* populations (Cluster 1 and Cluster 2). Interestingly, we observed shorter branches within the Cluster 6 clade than in Cluster 1. This pattern suggests that the prophages in Cluster 6 share a higher proportion of homologous groups or proteins with similar sequences, implying closer evolutionary relationships or less genetic divergence. In contrast, the prophages in Cluster 1, with longer branch lengths, appear to have undergone more divergence that could potentially result in a broader range of genetic content.

Additionally, G2 (Cluster 1) was primarily found in isolates from North America and South Asia, whereas the G7 (Cluster 6) *Vieuvirus* was mainly found in East Asia and Europe. These observations were validated by assessing the uncertainty coefficient, indicating that the identification of phage species is influenced by geographic location. In addition, some international clones (IC) of *A. baumannii* are known to be limited to specific geographic regions, and this localization is apparently timeless (Müller et al., 2023). For instance, IC9 is in the USA, whereas IC5 is in Latin America. Although it has been reported that similar lytic *A. baumannii* phages have been isolated in different geographic areas (López-Leal et al., 2021), prophage populations are geographically delimited (Tenorio-Carnalla et al., 2024).

Interestingly, of the 73 phage species that we found with a strong positive correlation between frequency and geographic co-occurrence, the species belonging to the genus *Vieuvirus* were the most prevalent (26.38%). Our results suggest that the geographic region and the circulating strains within that region could influence resistance or sensitivity to certain types of phages. Although phage and prophage detection tools may be biased by training data and genome availability, the regionality of prophage populations in *A. baumannii* has recently been reported (Tenorio-Carnalla et al., 2024). However, this has not been explored in other clinically relevant pathogens. This observation adds another factor to consideration in using phage therapy to combat MDR pathogens since prophages prevent superinfection.

Finally, our study provides valuable insights into the diversity and geographic distribution of prophages in *A. baumannii*. Identifying two geographically distinct populations of *Vieuvirus* highlights the need for further investigation into how phage-host interactions evolve in different regions. Moreover, our results underscore the importance of considering geographic factors and inducible prophage populations when developing phage therapy strategies, particularly in the context of MDR pathogens such as *A. baumannii*. Understanding the genetic diversity and distribution of prophages will be essential for advancing the use of bacteriophages as therapeutic agents in clinical settings.

## Data Availability

The datasets presented in this study can be found in online repositories. The names of the repository/repositories and accession number(s) can be found in the article/Supplementary material.

## Footnotes

^1^ https://www.bioinformatics.babraham.ac.uk/projects/trim_galore/

^2^ dx.doi.org/10.17504/protocols.io.bwm5pc86

## Supplementary material

SUPPLEMENTARY DATA 1 Blast results of isolated phage against the NCBI virus database.

SUPPLEMENTARY DATA 2 Agarose gel electrophoresis of restriction digestion products. Representative 1% agarose gel electrophoresis of restriction digestion products using *BamH*I/*Nde*I and *EcoR*I enzymes. Each gel lane corresponds to DNA from phage isolated from the same host (*A. baumannii* 5013 and/or *A. baumannii* 5038). The red arrows indicate the discriminatory bands each enzyme produces (or a mixture of enzymes), which indicates different phages.

SUPPLEMENTARY DATA 3 List of bacterial genomes used.

SUPPLEMENTARY DATA 4 CheckV report on the quality of viral sequences.

SUPPLEMENTARY DATA 5 List of phage genomes used from NCBI.

SUPPLEMENTARY DATA 6 Principal component analysis (PCA) was performed on a pan-matrix. Each phage genome is represented by a circle positioned in the pan-matrix space based on its values along the first two principal components. The colors of the circles indicate the phage clusters at the genus level. The percentages shown in the axis labels represent the total variation in the pan-matrix that is captured by each principal component. As shown in Figure 1 (see results), the circles indicate two distinct groups of phages: those that share several homologous groups (red oval) and those with few homologous groups (blue oval).

SUPPLEMENTARY DATA 7 Distributions of Cluster 1 **(A)** and Cluster 6 (right) branch lengths. The histograms show the observed branch length distributions for two clades (Cluster 1 and Cluster 6) from the tree in Figure 2.

SUPPLEMENTARY DATA 8 List of phages grouped in clusters at genus and species level.

SUPPLEMENTARY DATA 9 Table frequency of phages and prophages from the different genera (clusters) by host.

SUPPLEMENTARY DATA 10 Frequency of phages belonging to the genus *Vieuvirus* (Clusters 1 and 6) present in *A. baumannii* isolates from each geographic region.

SUPPLEMENTARY DATA 11 The hierarchical tree was constructed with the PC (protein clusters) of phages from cluster 13 based on intergenomic distances. The bootstrap values higher or equal to 80 are depicted with blue circles at the internal nodes. The color labels are found on the outer edge of the tree.
