# Supplementary material for: Geographic variation in abundance and diversity of Acinetobacter baumannii Vieuvirus bacteriophages
Source: Front Microbiol. 2025 Jan 28;16:1522711. doi: 10.3389/fmicb.2025.1522711 (PMC11813220; doi:10.3389/fmicb.2025.1522711)
Supplement: Supplementary file 1 [file Supplementary_file_1.zip › Supplementary Data 10.PDF]

Supplementary data 10.

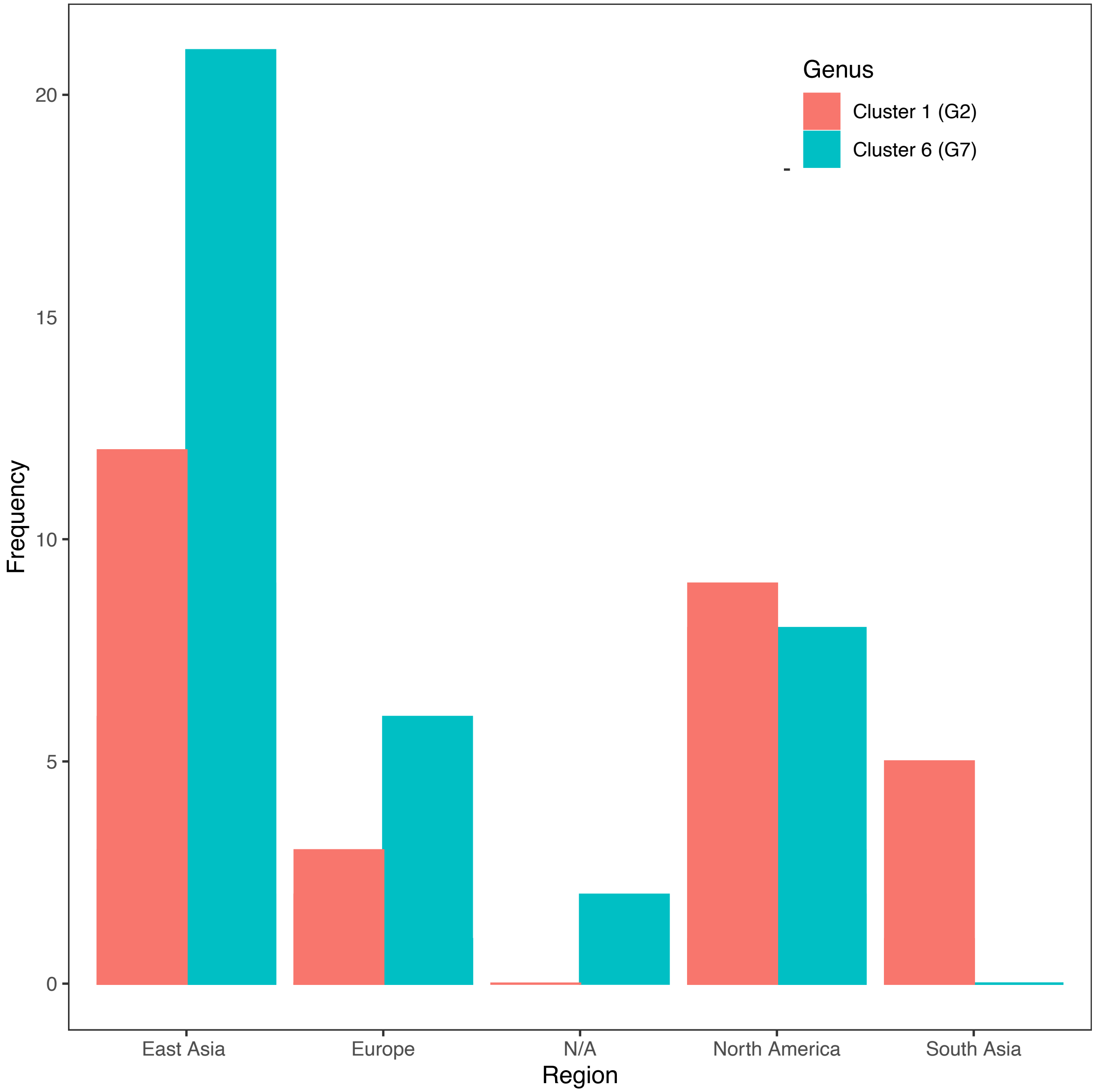

Frequency of phages belonging to the genus *Vieuvirus* (Clusters 1 and 6) present in *A. baumannii* isolates from each geographic region.
