# Supplementary material for: Geographic variation in abundance and diversity of Acinetobacter baumannii Vieuvirus bacteriophages
Source: Front Microbiol. 2025 Jan 28;16:1522711. doi: 10.3389/fmicb.2025.1522711 (PMC11813220; doi:10.3389/fmicb.2025.1522711)
Supplement: Supplementary file 1 [file Supplementary_file_1.zip › Supplementary Data 11.PDF]

Supplementary data 11.

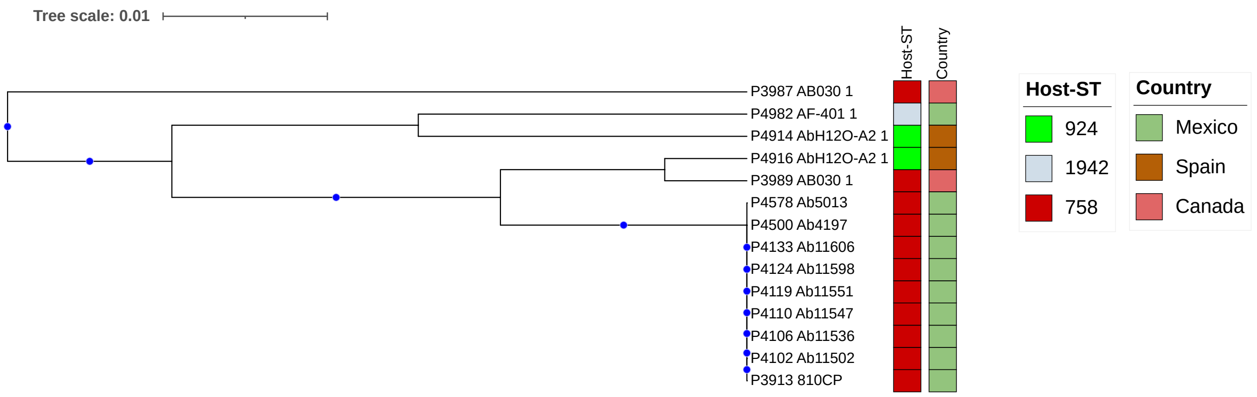

The hierarchical tree was constructed with the PC (protein clusters) of phages from cluster 13 based on intergenomic distances. The bootstrap values higher or equal to 80 are depicted with blue circles at the internal nodes. The color labels are found on the outer edge of the tree.
