# Supplementary material for: Geographic variation in abundance and diversity of Acinetobacter baumannii Vieuvirus bacteriophages
Source: Front Microbiol. 2025 Jan 28;16:1522711. doi: 10.3389/fmicb.2025.1522711 (PMC11813220; doi:10.3389/fmicb.2025.1522711)
Supplement: Supplementary file 1 [file Supplementary_file_1.zip › Supplementary Data 2.PDF]

Supplementary data 2.

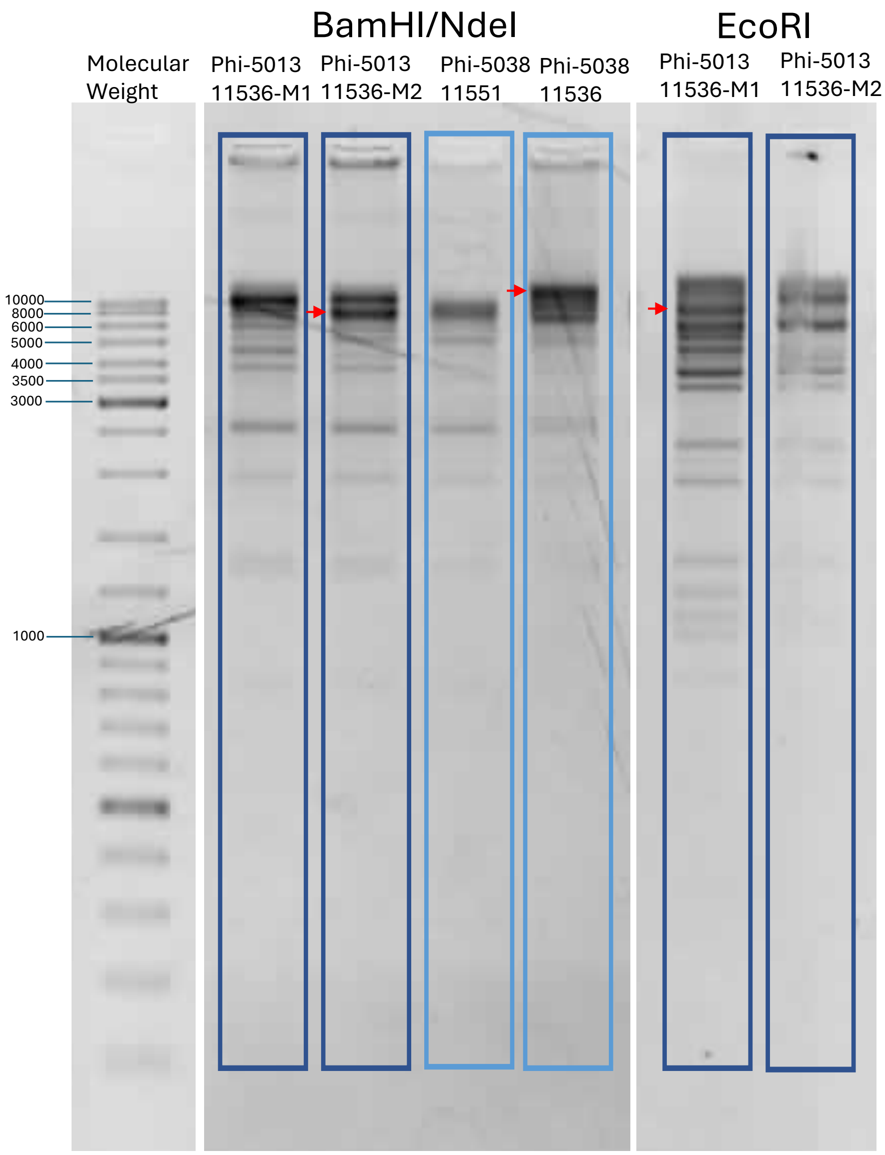

Agarose gel electrophoresis of restriction digestion products. Representative 1% agarose gel electrophoresis of restriction digestion products using *BamH*I/*Nde*I and *EcoR*I enzymes. Each gel lane corresponds to DNA from phage isolated from the same host (*A. baumannii* 5013 and/or *A. baumannii* 5038). The red arrows indicate the discriminatory bands each enzyme produces (or a mixture of enzymes), which indicates different phages.
