# Supplementary material for: Geographic variation in abundance and diversity of Acinetobacter baumannii Vieuvirus bacteriophages
Source: Front Microbiol. 2025 Jan 28;16:1522711. doi: 10.3389/fmicb.2025.1522711 (PMC11813220; doi:10.3389/fmicb.2025.1522711)
Supplement: Supplementary file 1 [file Supplementary_file_1.zip › Supplementary Data 6.PDF]

Supplementary data 6.

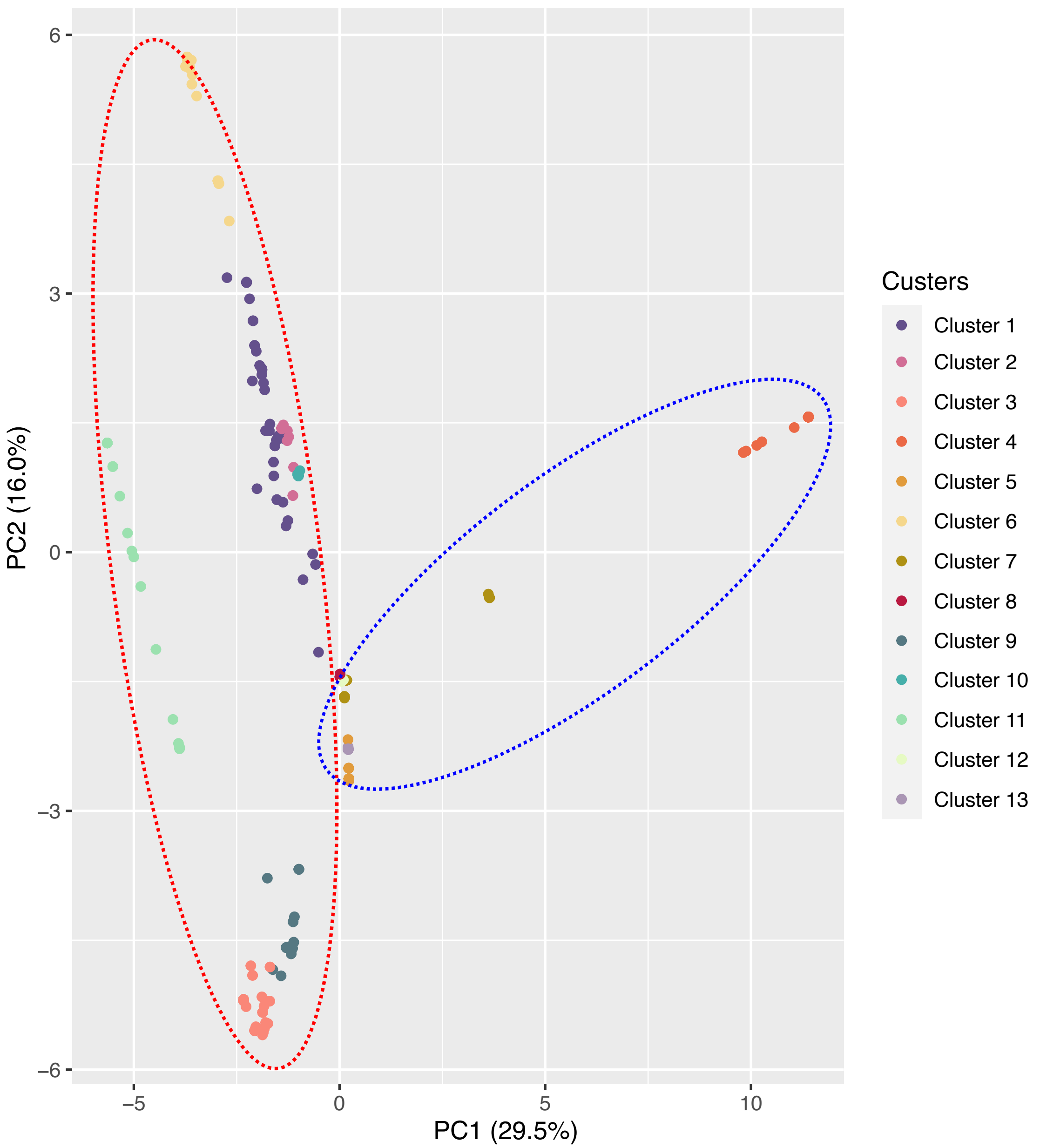

Principal component analysis (PCA) was performed on a pan-matrix. Each phage genome is represented by a circle positioned in the pan-matrix space based on its values along the first two principal components. The colors of the circles indicate the phage clusters at the genus level. The percentages shown in the axis labels represent the total variation in the pan-matrix that is captured by each principal component. As shown in Figure 1 (see results), the circles indicate two distinct groups of phages: those that share several homologous groups (red oval) and those with few homologous groups (blue oval).
