# Supplementary material for: Geographic variation in abundance and diversity of Acinetobacter baumannii Vieuvirus bacteriophages
Source: Front Microbiol. 2025 Jan 28;16:1522711. doi: 10.3389/fmicb.2025.1522711 (PMC11813220; doi:10.3389/fmicb.2025.1522711)
Supplement: Supplementary file 1 [file Supplementary_file_1.zip › Supplementary Data 7.PDF]

Supplementary data 7.

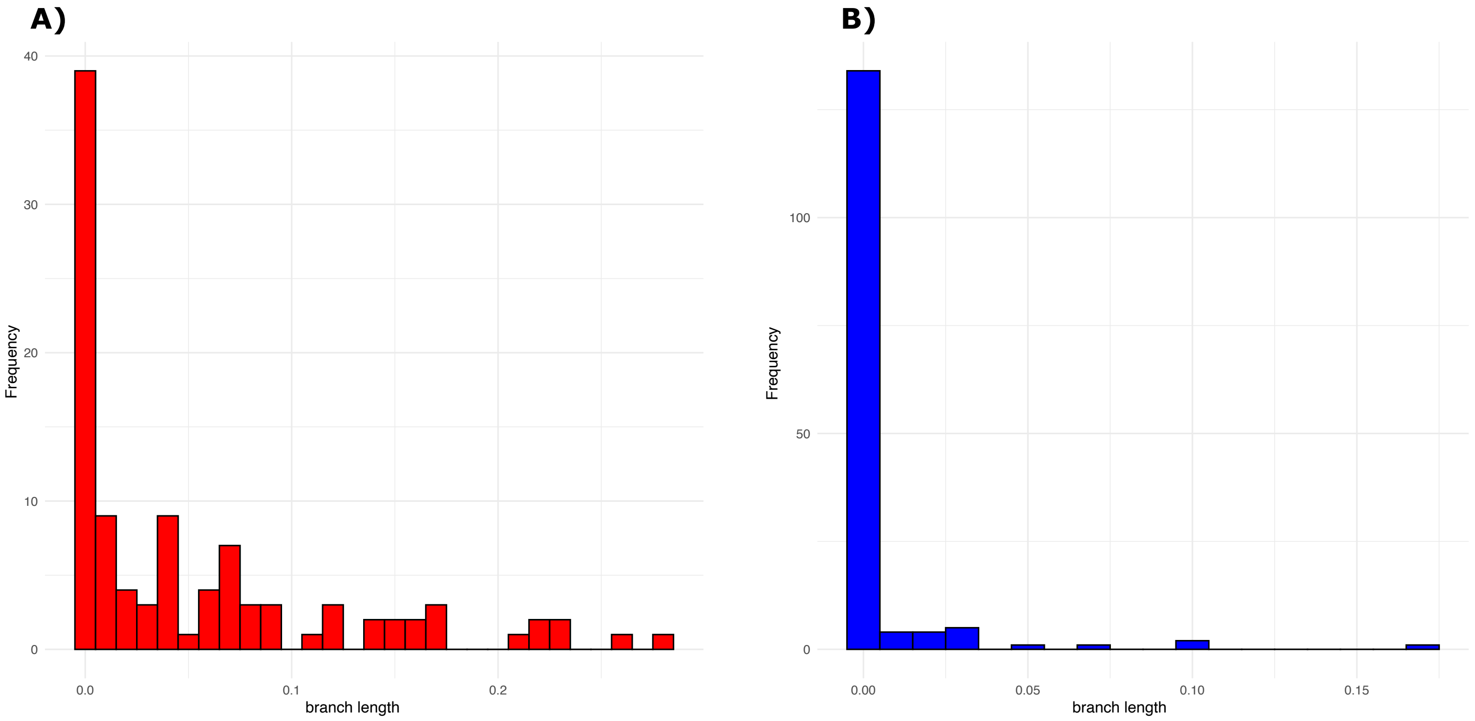

Distributions of Cluster 1 (A) and Cluster 6 (right) branch lengths. The histograms show the observed branch length distributions for two clades (Cluster 1 and Cluster 6) from the tree in Figure 2.
